# Technology for Alzheimer's and Dementia diagnosis in Latin America (LatAm): Challenges and opportunities

**DOI:** 10.1002/alz70858_107189

**Published:** 2025-12-26

**Authors:** Eyitomilayo Yemisi Babatope, Alejandro Álvaro Ramírez‐Acosta, Mireya Saraí García‐Vázquez

**Affiliations:** ^1^ Instituto Politécnico Nacional, CITEDI, Tijuana, BJ, Mexico; ^2^ MIRAL R&D&I Multimedia, San Diego, CA, USA

## Abstract

**Background:**

Globally, technology is a powerful transformative tool, with undeniable innovations and record‐breaking potential in almost every sector. Advancement in technology has paved the way for improvement in Alzheimer's disease (AD) and other types of dementia. Technologies including artificial intelligence driven approaches, wearable devices, smart home technologies, computerized batteries are used for health monitoring and diagnosis. In Latin American countries, the growing research effort is evident in the annual satellite symposium organized by the Alzheimer's association. This review evaluates various existing technological tools that are used in the Latin American countries for the diagnosis of Alzheimer's disease.

**Method:**

We conducted a rigorous search of data repositories such as web of science, Scopus, PubMed, science direct and Wiley online library to identify papers related to the use of technology in AD diagnosis within this region. Keywords such as assistive technology, smart home technology, artificial intelligence, Alzheimer's disease, dementia, diagnosis, monitoring, and support, were used. The selected paper will be analyzed, and the findings discussed.

**Result:**

Evidence from the selected articles will be summarized and presented to emphasize the role of these technological tools in Alzheimer's diagnosis within the Latin American countries. The challenges identified by the authors of these papers will be discussed.

**Conclusion:**

Although these technologies offer promising solutions, the challenges associated with their use in these Latin American countries will be discussed to bridge the gap and provide solutions to overcome these challenges. This review evaluates various existing technologies with the intention to interdisciplinary collaboration between end users (the clinicians providing care, the people living with the disease, caregivers and family members) and technology experts to optimize its impact.

